# Liposomes in Drug Delivery: How It All Happened

**DOI:** 10.3390/pharmaceutics8020019

**Published:** 2016-05-24

**Authors:** Gregory Gregoriadis

**Affiliations:** UCL School of Pharmacy, 29-39 Brunswick Square, London WC1N 1AX, UK; gregoriadis@xeneticbio.com

Effective delivery of drugs via liposomes in the treatment or prevention of disease is the aim of numerous researchers worldwide. Therapies include those for cancer, microbial infections, hormone and enzyme deficiencies, metal detoxification, gene deficiency or malfunction, as well as vaccines. Intimate knowledge of the structure and physical properties of liposomes, and of ways such properties influence their behaviour within the biological milieu, is central to their success.

The discovery of liposomes in the mid-1960’s [1] and their similarity to cell membranes presented cell biologists with a unique tool for the study of a number of cell membrane functions including cell fusion, membrane pumps and antigen presentation. However, it was not until several years later that liposomes were considered as a candidate carrier for the delivery of pharmacologically active agents in the treatment of disease [2,3,4].

The use of liposomes in drug delivery and targeting is often discussed in the context of decades marked by significant milestones. Thus, the 1970s are noted for the initial understanding of the system’s behaviour *in vivo*, namely its interaction with the biological milieu in the living animal and, as a result, the proposition of an array of therapeutic applications. Following a period of “disillusionment” of those (mainly in industry) with expectations unjustified by the degree to which liposomes had been developed at that time, the 1980s were a period of reflection and consolidation. This was also a period of advancements in improving liposomal stability in biological fluids such as blood [5], in liposome technology [6] in terms of developing techniques for high yield entrapment, and the preservation of intact liposomes under storage. Importantly, the founding in 1981 of three liposome-based companies in the USA ensured a systematic transition of some of the earlier concepts [7,8,9] into realistic goals backed by significant progress in large-scale technology. The 1990s were clearly the decade of clinical trials, approved injectable products (e.g., AmBisome, Doxil), and of new horizons. Forty six years after work on the use of liposomes in drug delivery commenced, enthusiasm is still prevailing. The old guard of liposomologists who were ‘there’ from the very beginning, but are gradually retiring into new pastures, are being replaced by worthy successors. Below I discuss early developments which are thought to have helped shape the future of the field. My audience includes young liposomologists entrapped in the maze of a myriad of publications of varying clarity, insight, accuracy and, perhaps, bias.

My involvement with liposomes, described in more detail elsewhere [10] began with a chance event. In 1969, while in New York, I came across a Nature advertisement for a research post with the late Brenda Ryman (Figure 1) on the delivery of enzymes to the hepatic parenchymal cells. I was attracted by it because of my work [11,12] at the Albert Einstein College of Medicine, on the discovery of the hepatic galactose receptor and the opportunity presented by the post to pursue galactose-terminating ligands in targeting drugs to the liver. On arrival in Ryman’s laboratory in the summer of 1970, it turned out that one of the candidate systems for enzyme delivery was one called “liposomes”. Other systems also considered were nylon micro capsules and solid support systems. It was obvious, however, even in those early days, that the expected, innocuous, non-toxic biodegradable nature of liposomes, their sub-micrometre size and apparent structural versatility rendered them the candidate of choice. Because of my familiarity with animal work in previous years on the fate of macromolecules (glycoproteins) *in vivo* at the cellular and subcellular level, facts about the fate of intravenously injected liposomes and entrapped contents were easy to establish. We were able to show [3,4], that liposomes can deliver enzymes into the lysosomes of the tissues of the reticular endothelial system (RES), the very place where lipids, polysaccharides and other molecules accumulate in lysosomal storage conditions. Thus, our data supported the effective use of liposomes in enzyme replacement therapy. I was able to confirm this later in a model lysosomal storage disease [13]. It was 1972, our work had been acknowledged by A. D. Bangham and his colleagues (Figure 2), the territory in the use of liposomes as a delivery system in therapeutics was uncharted and we were raring to go, albeit in our separate ways. Brenda was appointed to a Chair in the Charing Cross Medical School and I on the staff of the Medical Research Council’s Clinical Research Centre at Harrow.

Being aware of the need for specific drug action in a multitude of therapies, exploration of the potential uses of liposomes was extended to cancer and antimicrobial (intracellular) therapy [14,15,16], and established [17,18] the concept of vesicle targeting with surface-bound antibodies and other cell-specific ligands (e.g., asialoglycoproteins). Equally exciting but perhaps more significant in its implications was the finding [19,20,21] that liposomes potentiate immune responses to entrapped protein antigens. Seen from today’s perspective, these forty-odd-year-old papers would appear courageously naïve in their claims, and vulnerably assertive in their optimism. Yet, those innocent flights of fancy have ended up, as stated elsewhere [10], “wrapped in red ribbons on the desks of hard-nosed lawyers, eagle-eyed patent attorneys and worried CEO’s, or hidden in the highs and lows of the NASDAQ stock list.”

A major potential disadvantage of the liposomal carrier is that, following intravenous injection, it is rapidly intercepted by the fixed macrophages of the liver and spleen. However, the involvement of the RES in vesicle uptake is the basis of the mode of action of several of the licensed liposome-based products, including vaccines against Hepatitis A and influenza. Added to this is the promise [22,23] of liposome-based DNA vaccines, especially when the plasmid DNA is co-entrapped in the same liposomes together with the protein antigen it encodes [24]. On the other hand, a significant delay of RES participation in the uptake of liposomes would prolong their time of circulation in the blood, thus enabling them to reach and deliver their drug content to alternative tissues thus enlarging the spectrum of possible therapies. The way by which the challenge of long circulating liposomes was met is one of the better examples of rational system design. It was based on the use of neutral small unilamellar vesicles known [25] to persist in the blood circulation for much longer periods of time than similar, charged vesicles or larger (multilamellar) liposomes regardless of surface charge. We found that the addition of (equimolar to the phospholipid) cholesterol [26] in the bilayers and the use of high-melting phospholipids [27,28], also observed independently by Hwang *et al.* [29], led to vesicles that were resistant to the destabilising action of plasma high density lipoproteins [28]. It turned out that the greater the stability of liposomal bilayers in terms of entrapped solute retention in the presence of blood serum, the greater the half-life of liposomes in the circulating blood [28]. Consequently, most marketed liposome-based injectable products consist of high-melting phospholipids and equimolar cholesterol. A subsequent, equally important, innovation by several groups, including the author’s [30,31,32,33,34], also contributed to extending the circulatory half-life of liposomes by coating their surface with polyethylene glycol, known for its ability to interfere with opsonin adsorption on the vesical surface and vesicle recognition by the RES.

Such is the structural versatility of the liposomes, it renders the design of vesicle versions destined for specific needs practically limitless. In this respect, newer developments [35] include the design of liposomes for tumour targeting, gene and siRNA therapy, genetic vaccines, immunomodulation, as well as a variety of transdermal applications [36]. The reader will have noted the author’s optimism for the longer term future of the liposomal carrier. One might have considered the difficulty of inventing an alternative carrier of similar attributes and potential. One might have dreamed of possible ruses in molecular modelling to circumvent the impossibility of disciplining every drug for erratic or dangerous behaviour once allowed in the body. If so, one might then understand the reasons of the author’s chronic addiction to the system he happened to come across so many years ago.

## Figures and Tables

**Figure 1:**
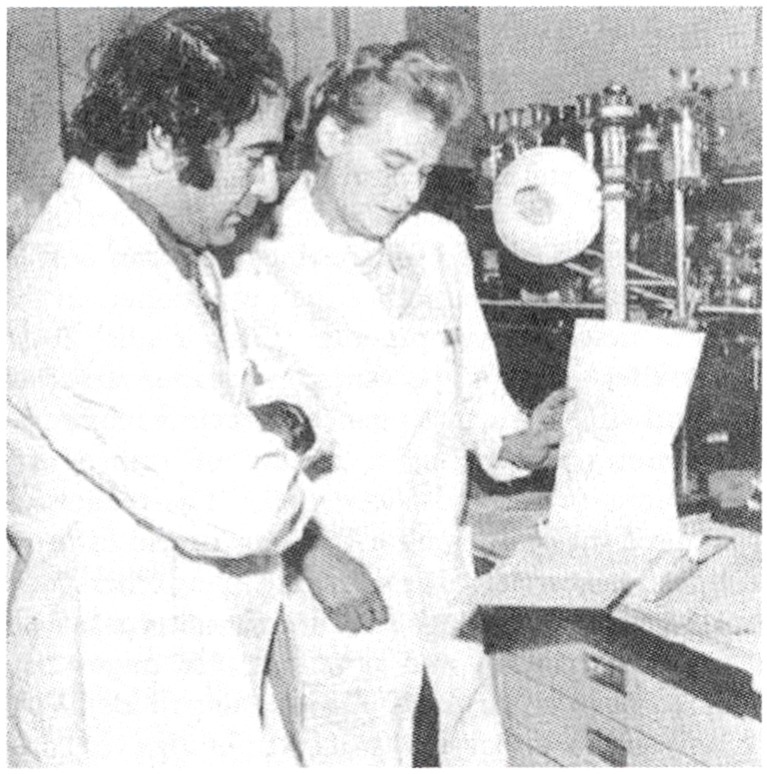
Brenda Ryman with the author (*circa* 1971).

**Figure 2:**
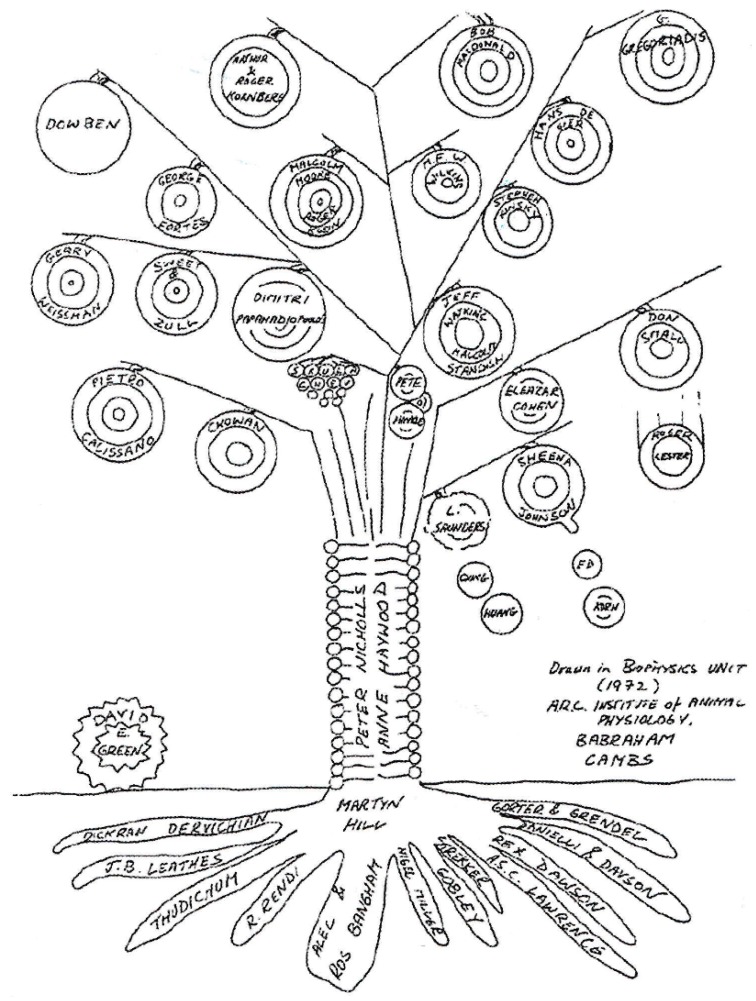
Participants in the evolution of liposomes. Drawn in 1972 at the Unit of Biophysics, ARC Institute of Animal Physiology, Babraham.
